# Integrative analyses of gene expression profile reveal potential crucial roles of mitotic cell cycle and microtubule cytoskeleton in pulmonary artery hypertension

**DOI:** 10.1186/s12920-020-00740-x

**Published:** 2020-06-26

**Authors:** Jing Luo, Haiyan Li, Zhenwei Liu, Chenlu Li, Ruochen Wang, Jinxia Fang, Saisai Lu, Jing Guo, Xiaochun Zhu, Xiaobing Wang

**Affiliations:** 1 Rheumatology Department, The First Affiliated Hospital of Wenzhou Medical University, Wenzhou, 325035 China; 2 Department of Pediatric Pulmonology, The Second Affiliated Hospital and Yuying Children’s Hospital of Wenzhou Medical University, Wenzhou, China; 3 Institute of Genomic Medicine, Wenzhou Medical University, Wenzhou, China; 4 College of psychologic medicine, Wenzhou Medical University, Wenzhou, China

**Keywords:** Pulmonary arterial hypertension, Differentially expressed gene, Functional enrichment analysis, Protein-protein interaction network, miRNAs

## Abstract

**Background:**

Pulmonary arterial hypertension (PAH) is a life-threatening condition. The aim of this study was to explore potential crucial genes and pathways associated with PAH based on integrative analyses of gene expression and to shed light on the identification of biomarker for PAH.

**Methods:**

Gene expression profile of pulmonary tissues from 27 PAH patients and 22 normal controls were downloaded from public database (GSE53408 and GSE113439). After the identification of differentially expressed genes (DEGs), hub pathways and genes were identified based on the comprehensive evaluation of protein-protein interaction (PPI) network analysis, modular analysis and cytohubba’s analysis, and further validated in another PAH transcriptomic dataset (GSE33463). Potentially associated micro-RNAs (miRNAs) were also predicted.

**Results:**

A total of 521 DEGs were found between PAH and normal controls, including 432 up-regulated DEGs and 89 down-regulated DEGs. Functional enrichment analysis showed that these DEGs were mainly enriched in mitotic cell cycle process, mitotic cell cycle and microtubule cytoskeleton organization. Moreover, five key genes (*CDK1, SMC2, SMC4, KIF23,* and *CENPE*) were identified and then further validated in another transcriptomic dataset associated with special phenotypes of PAH. Furthermore, these hub genes were mainly enriched in promoting mitotic cell cycle process, which may be closely associated with the pathogenesis of PAH. We also found that the predicted miRNAs targeting these hub genes were found to be enriched in TGF-β and Hippo signaling pathway.

**Conclusion:**

These findings are expected to gain a further insight into the development of PAH and provide a promising index for the detection of PAH.

## Background

Pulmonary arterial hypertension (PAH) is a severe chronic and progressive vascular disorder, predominantly influencing the arterial circulation and, in particular, the pulmonary arterioles [1]. PAH is defined based on the elevation of the mean pulmonary arterial pressure (mPAP) above 25 mmHg at resting state and a pulmonary vascular resistance > 3 Wood units, as well as a pulmonary capillary wedge pressure (PCWP) < 15 mmHg at end expiration [2]. However, this 6th World Symposium on Pulmonary Hypertension (WSPH) Task Force proposed to define the low limit of mPAP for PAH to be 20 mmHg [3] The increase in afterload puts great stress on the right ventricle (RV), leading to RV hypertrophy, and ultimately RV failure and death [4]. It has a prevalence of about 20 cases in 1,000,000 population and particularly affects women with four times more than men [5]. According to latest classification criteria, PAH can be divided into idiopathic PAH (IPAH), heritable PAH, drug- and toxin-induced PAH, PAH associated with other disease (connective tissue disease, HIV infection, portal hypertension, congenital heart disease, schistosomiasis) and so on [3]. In the past several decades, lots of studies have been undertaken to clarify the mechanism of the progression of PAH and apply new promising target therapy, while the incidence and mortality rate of PAH remains high with poor prognosis [6, 7]. Hence, revealing the causes and underlying molecular mechanisms of the disease, as well as discovering molecular biomarkers for early diagnosis, prevention and personalized treatment, is especially important and highly demanded for PAH.

Microarray has been used to detect massive genes expression for more than 10 years, and is particularly suitable for DEGs screening [8]. With the extensive using of microarray technology, an increasing number of chip data were produced and deposited into public databases including the largest public database: NCBI-Gene Expression Omnibus (NCBI-GEO) database. Several microarray studies have been conducted on PAH in recently years and hundreds of DEGs have been obtained [9, 10]. However, most analytical results are inconsistent among these studies mainly due to sample heterogeneity and study design. Thus, there has been no unified biomarker and commonly accepted biological mechanism acquired from these microarray studies for PAH.

Therefore, in our study, we obtained two original microarray datasets GSE53408 and GSE113439 (unpublished data) with the same platform of GPL6244 from GEO database [11, 12]. After merging the two datasets based on the same platform, we performed comprehensive biological functional analyses of DEGs from various angles, including Gene Ontology (GO) enrichment, pathway enrichment, protein-protein interaction (PPI) network and prediction of correlative miRNAs, which could largely overcome the disadvantages of previous single array studies. More importantly, identifying DEGs with their biological functions and key pathways will assist with providing more accurate and reliable biomarkers for early diagnosis of PAH.

## Methods

### Data attainment and preprocessing

The gene expression data of PAH were obtained from the NCBI-GEO database (http://www.ncbi.nlm.nih.gov/geo/). Respectively, two GEO series (GSE53408 and GSE113439) were chose in our study with the following selection criteria: (a) keywords of “pulmonary artery hypertension (PAH)” or “pulmonary hypertension (PH)”; (b) Inclusion of gene expression data of PAH and normal tissue samples with the same GEO platform; (c) excluding other diseases except PAH and normal tissues, such as pulmonary fibrosis or interstitial pneumonia (d) Datasets contained a minimum of 10 PAH and normal tissue samples and inclusion of > 5000 genes in the GEO platform.

The raw data were manipulated with the process of background adjustment, quantile normalization, logarithmic transformation and summarization by using the “Affy” package of R language [13]. Afterwards, according to the annotation files provided by GPL6244, the expression matrix with the probe IDs were converted into gene symbols. The “Impute.Knn” function of “impute” package was applied to supplement missing value [14] and probes without a corresponding gene symbol were deleted and the average value was calculated as the final expression value for genes corresponding to more than one probe. Moerover, the “ComBat” function of “sva” package was used to remove known batch effects from microarray data [15] and quantile normalization within and between arrays on all samples was conducted using “normalizeBetweenArrays function” function.

### Identification of differentially expressed genes

We used the limma package [16] to implement DEGs analysis, and used “princomp” function in R 3.6.0 to conduct a two-dimensional principle component analysis (PCA) and hierarchical clustering to visualize the similarities, as well as the differences between the PAH and the control samples. Subsequently, differential expression analysis was performed and a DEG was defined based on the following criteria: *p* value< 0.05 and the absolute value of log_2_ fold change (FC) > 1. The volcano plot, which visualized all DEGs between PAH and control, was performed with “ggplot2” package in R and clustering heatmap for the DEGs was drawn using the R software package “pheatmap”.

### Functional analysis of DEGs

To investigate the biological function of DEGs in PAH, we conducted gene ontology (GO) analysis including biological processes (BP), cellular components (CC), and molecular functions (MF). “ClueGo” plug-in [17] integrated GO terms in Cystoscape, and created biological process networks with the up-regulated and down-regulated genes, respectively. Bonferroni step down method was used for correction and the threshold of *p*-value was 0.05. Significant pathway analysis was conducted by the function “Gene-list Enrichment” with same threshold of p-value in online websites of Kobas3.0 (http://kobas.cbi.pku.edu.cn) including four signaling pathway analysis: KEGG Pathway, Reactome, BioCyc, and PANTHER.

### Identification and validation of hub genes

The PPI data of the DEGs was downloaded from STRING version 11.0. Then, the PPI network was set up and visualized by Cytoscape [18] software, and hub genes were detected according to levers of degree (the number of connections/interactions for each node) in PPI network. To further validate the key genes, the plug-in MCODE [19] was used to find out several functional modules based on MCODE score, which represented the degree of interrelation of DEGs. Subsequently, we also used plug-in cytoHubba of Cytoscape to explore important nodes in the PPI network by several topological algorithms including Betweenness, Bottle Neck, Closeness, Clustering Coefficient, Degree, DMNC, EcCentricity, EPC, MCC, MNC, Radiality and Stress [20]. The top 50 genes identified by each topological algorithm were selected to found the shared genes more than 6 ways as the most important hub genes in the network [21]. Finally, the shared hub genes detected by three methods (levers of degree in PPI network, MCODE score and topological algorithms in cytoHubba) were identified as the pivotal genes.

The transcriptomic data set of peripheral blood mononuclear cells (PBMCs) (GSE33463, Table S1), includes 30 IPAH, 19 patients with systemic sclerosis (SSc) without pulmonary hypertension, 42 scleroderma-associated PAH patients (SSc-PAH), and 8 patients with SSc complicated by interstitial lung disease and PH (SSc-PH-ILD), which were used to validate DEGs identified compared with 41 healthy individuals. By further analyzing expressional levers of pivotal genes with Wilcoxon and Kruskal−Wallis test, hub genes were validated and were exhibited as Violin diagram using R software package “ggpubr”. Subsequently, correlation analysis of hub genes was conducted by R software package “ggcorrplot”. To validate the function of hub-genes in distinguishing PAH cohorts from the control groups, the clustering heatmap for hub genes was drawn using the function “heatmap.2” of R software package “gplots”.

### Identification of hub genes associated with respiratory tract diseases

The Comparative Toxicogenomics Database (CTD; http://ctdbase.org/), a premier public resource based on literature, was used to find curated associations between chemicals interactions, gene interactions, phenotypes, diseases, and environmental exposures. In the database, the Inference Score was calculated from original source articles to present the relationship of genes to diseases. Here, we used the CTD database to analyze the associations between hub genes and respiratory tract diseases, and identified their relationships based on ranks of Inference Score.

### Prediction of miRNAs interacted with hub genes and function analysis

The miRNA-mRNA (hub genes) interaction networks were predicted based on Diana-microT-CDS (http://www.microrna.gr/microT-CDS/), TargetScan (http://www.targetscan.org/), miRDB (http://www.mirdb.org/) and mirDIPsoftware (http://ophid.utoronto.ca/mirDIP/) respectively. By setting “threshold as 0.7” in microT-CDS, “score class as very high (top1%) or high (top5%)” in mirDIP and “Total context ++ score” ≤ − 0.2 in TargetScan, we identified the intersection of four database as prediction of miRNAs for each hub gene. Cytoscape was applied to visualize the miRNA-mRNA interaction network and bubble diagram was selected for exhibiting function analysis of miRNA using online tools from Diana-miRPath v3.0 (http://www.micro rna.gr/miRPathv3).

## Results

### DEGs in subgroups of PAH

Fig.S1 showed the workflow for identification, functional analysis and validation of DEGs in PAH. To get a list of PAH-related DEGs, we compared the gene expression profiles of lung tissues of PAH patients with samples from healthy volunteers. Based on the GPL6244 [HuGene-1_0-st] Affymetrix Human Gene 1.0 ST Array, the microarray data included 12 cases and 11 control samples from GSE53408, as well as 15 cases and 11 control samples from GSE113439 (Table S1).

**Fig. 1:**
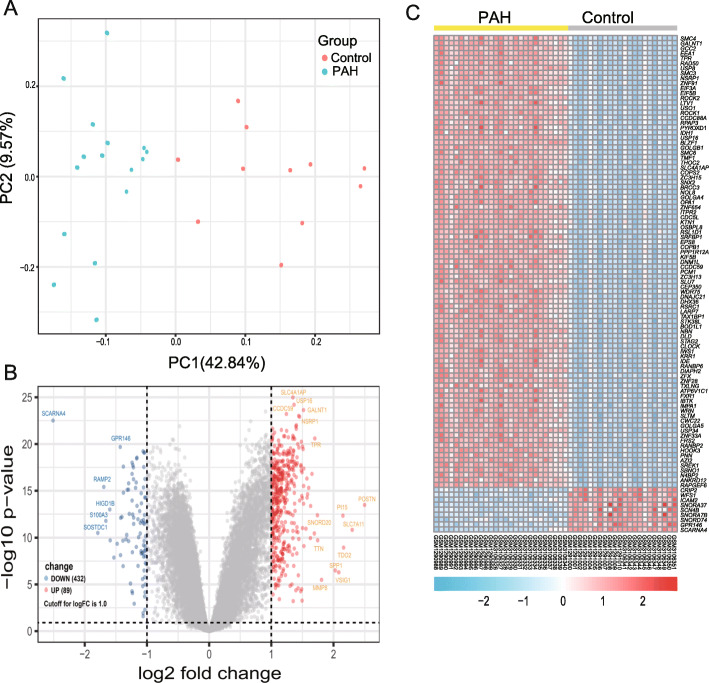
Differential expression analyses for all genes between PAH and control. **a.** Principal component analysis (PCA) illustrated the individual differences in the Chip expression profiles among the PAH and control groups in 49 samples. The green and red circles represent the case and control groups, respectively. The first component represent 42.84% of variance and the second component represent 9.57% of variance. The PCA plot was plotted using prcomp function in R program. **b.** Volcano plot showed all the gene expression change in PAH compared to the control samples. Grey represents no change in expression, blue represents downregulation (Down), and red represents upregulation (Up). Log FC reprsents log2 fold changes and *p*-value < 0.05 was considered as the threshold value of significant difference. The cutoff of log FC was 1.0. **c.** Heatmap showed the top 100 differentially expressed genes listed by listed by corrected *P*-values in PAH compared to the control samples. Each column represents one sample, and each row represents one gene. The gene expression values of all samples are showed as base − 2 logarithmic value. The gradual color ranging from blue to red represents the changing process from downregulated to upregulated expression

We undertook quality control of these datasets, and observed that gene expression distribution of each sample from the two different resources were homogeneous and comparable after data preprocessing (Fig.S2 A). PCA analysis revealed that PAH samples and control were clearly separated into two distinct clusters (Fig. 1a), indicating the discriminative gene expression pattern of PAH. Based on the cut-off criteria (*p* < 0.05 and [logFC] > 1), 521 DEGs between PAH and normal controls were identified, including 432 up-regulated genes and 89 down-regulated genes displayed by volcano plot (Fig. 1b and Table S2). The expression of the top 100 DEGs listed by corrected *P*-values was depicted by heatmap (Fig. 1c), from which we can see significant different clustering between two groups.

### Functional enrichment analysis of DEGs

**Fig. 2:**
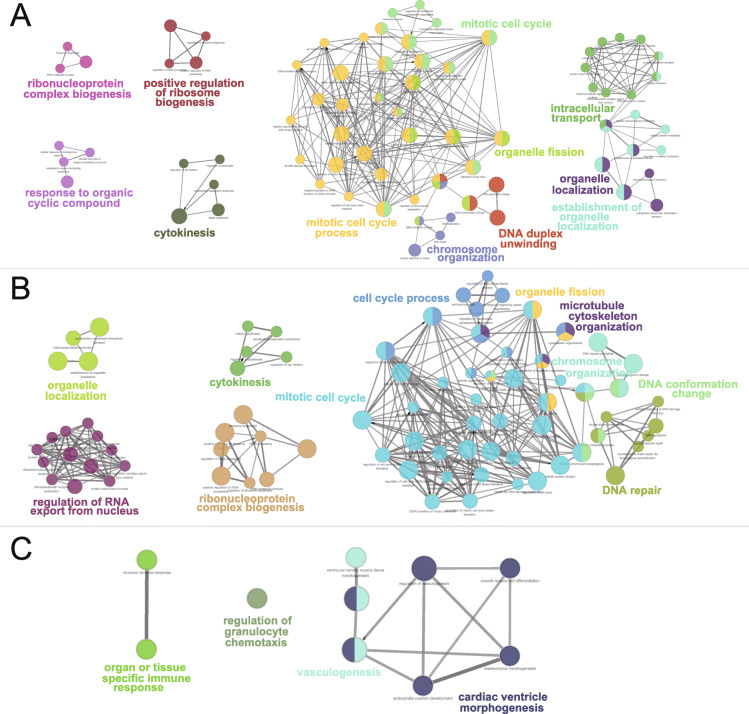
GO term enrichment analysis for DEGs revealed several biological processes associated with PAH**.** ClueGo network analysis was performed for all differentially expressed genes(**a**), up-regulated DEGs (**b**) and down-regulated DEGs (**c**). Enrichment for GO groups was conducted using the ClueGO plug-in. Nodes were colored according to grouping of related functions by statistically significant association of related GO terms. In each group, only the most significant term was labeled, and the node size corresponded to the significance of each GO term

To further interpret the biological processes associated with DEGs, ClueGo plug-in for Cytoscape was used to cluster GO terms that participate in the same biological function and visualize of the interactions inside each cluster, as well as between different groups. As shown in Fig. 2a and Table S3, the DEGs were significantly enriched in several biological processes (BP) potentially associated with PAH including mitotic cell cycle (GO: 0000278), mitotic cell cycle process (GO: 1903047) and microtubule cytoskeleton organization (GO: 0000226), all of which play essential roles in cell proliferation. As for cell component (CC), we found that candidate DEGs were mainly enriched in intracellular non-membrane-bounded organelle (GO:0043232), nuclear lumen (GO:0031981) and microtubule cytoskeleton (GO:0015630). In addition, the candidate DEGs were significantly enriched in ATP binding (GO: 0005524), adenyl ribonucleotide binding (GO: 0032559) and adenyl nucleotide binding (GO: 0030554) in the molecular function (MF) group.

Moreover, we also investigated the biological processes of all up-regulated and down-regulated genes which were exhibited severally in Fig. 2b and c, respectively. We found that up-regulated genes were also mainly enriched in cell cycle process (GO: 0022402) and mitotic cell cycle process (GO: 1903047) highly consistent with all DEGs. Nevertheless, down-regulated genes were mainly enriched in vasculogenesis (GO:0001570) and regulation of vasculogenesis (GO:2001212) with unapparent interaction. In summary, the GO analyses indicated that most of DEGs were significantly enriched in mitotic cell cycle process, microtubule cytoskeleton organization, intracellular non-membrane-bounded organelle, and ATP binding, all of which are associated with the cell proliferation [22, 23]. As shown in Table S4, pathway enrichment analysis showed that cell cycle, metabolism of proteins and cell cycle, mitotic were top 3 significantly enriched pathways according to the corrected *P*-Value, which were also consistent with GO term enrichment analysis.

### Protein–protein interaction network (PPI), modular and CytoHubba analysis

**Fig. 3:**
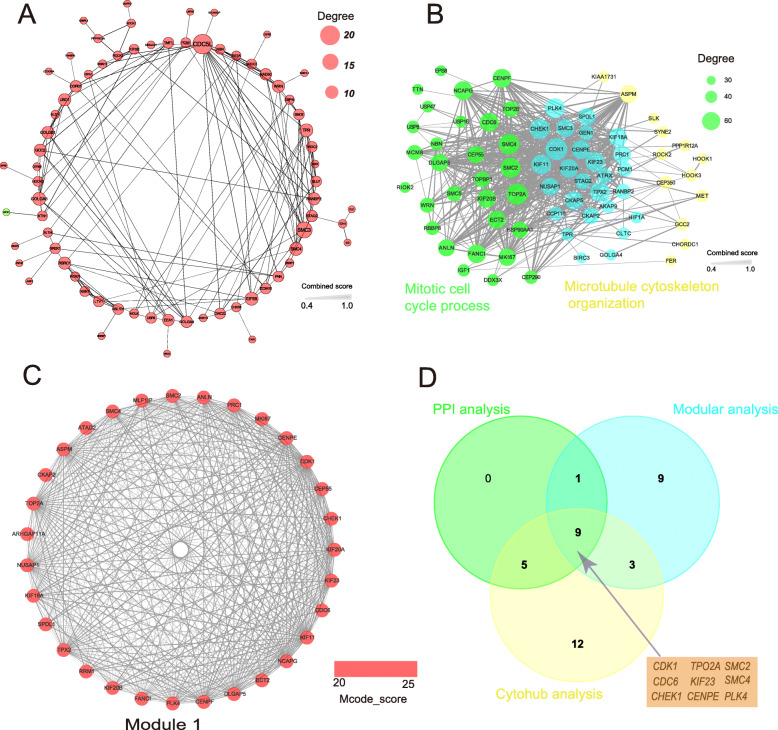
Identification of hub genes from DEGs. **a.** The protein–protein interaction (PPI) network of top 100 DEGs. Red nodes represent up-regulated DEGs and green nodes represent down-regulated DEGs. The size of circles represents levers of degree (number of interaction) and the thickness of edge represent strength of interaction with combined score. **b.** PPI network of the genes enriched in Mitotic Cell Cycle and Microtubule Cytoskeleton Organization. The nodes represented genes, blue indicated the DEGs shared by the two biological processes and the other colors corresponded to the genes in these two biological processes. And the size of nodes indicated the number of connections. Edges denoted the interactions between two genes, and the width of an edge denoted the score of a physical interaction. **c.** The core module (module 1 with the MCODE score of 29.067) from the PPI network. The color shadow of each node represents the Mcode_score (degree of connection of nodes). **d.** Venn diagram indicated 9 hub genes overlapping from three analytic procedure (PPI, Modular and Cytohub)

To identify central attractors for DEGs in the physical interaction network and provide clues for further pathogenic mechanism of PAH, we constructed an interconnected PPI network of DEGs based on the Search Tool for the Retrieval of Interacting Genes (STRING) database (https://string-db.org/). A total of 521 DEGs were filtered into the network, forming 492 nodes and 2720 edges with average node degree of 11 (Fig.S3). Among the 492 nodes, the top 15 node genes listed by the number of connections were identified with degree of larger than 40 (each node had more than 40 connections/interactions), including *TOP2A, TOP2B, CDK1, LRRK2, HSP90AA1, EPRS, POLR2B, SMC2, CHEK1, SMC4, KIF23, PLK4, KIF11, CDC6* and *CENPE* (Table S5). In the PPI network of top 100 ranked DEGs according to the *P*-values, we found that up-regulated genes have more significantly close interaction than down-regulated genes (Fig. 3a). Given that mitotic cell cycle process indicated the most prominent features in up-regulated DEGs based on GO term enrichment and microtubule cytoskeleton organization especially in microtubule had been found exist important interaction with PAH through text mining [24], we performed the interconnected interaction network of DEGs among these two biological processes. We found that there were 70 DEGs with a close interconnected relationship and 27 DEGs shared by these two biological processes (Fig. 3b).

For further modular analysis for DEGs in the PPI network, we identified 3 significant modules from the PPI network complex by using MCODE with MCODE score greater than 5 (Fig. 3c and Fig.S2 B). Pathway enrichment analysis using Kobas3.0 showed that Module 1, consisted of 31 nodes and 446 edges, were mainly associated with cell cycle, cell cycle mitotic and M phase. For Module 2, it was consisted of 20 nodes and 173 edges, which were mainly associated with major pathway of ribosomal RNA (rRNA) processing in the nucleolus and cytosol, as well as ribosome biogenesis in eukaryotes. In addition, the Module 3 were mainly associated with cellular response to heat stress, regulation of HSF1-mediated heat shock response and protein processing in endoplasmic reticulum, including 17 nodes and 65 edges (Table S6). Based on the levers of MCODE score, we identify 22 hub genes with largest node’s MCODE score 24 in Module 1(bold in Table S5) [25].

In addition to aforementioned two functional analysis, hub genes were also identified by CytoHubba analysis. We selected the top 50 key genes identified from each method of CytoHubba and found that there were 29 hub genes shared with more than 6 topological analysis methods (Table S7). Summarily, there were 9 key genes shared by all three analysis, including *TOP2A, CDK1, SMC2, CHEK1, SMC4, KIF23, PLK4, CDC6* and *CENPE* (Fig. 3d).

### Validation of hub genes and identification of correlative respiratory tract diseases

**Fig. 4:**
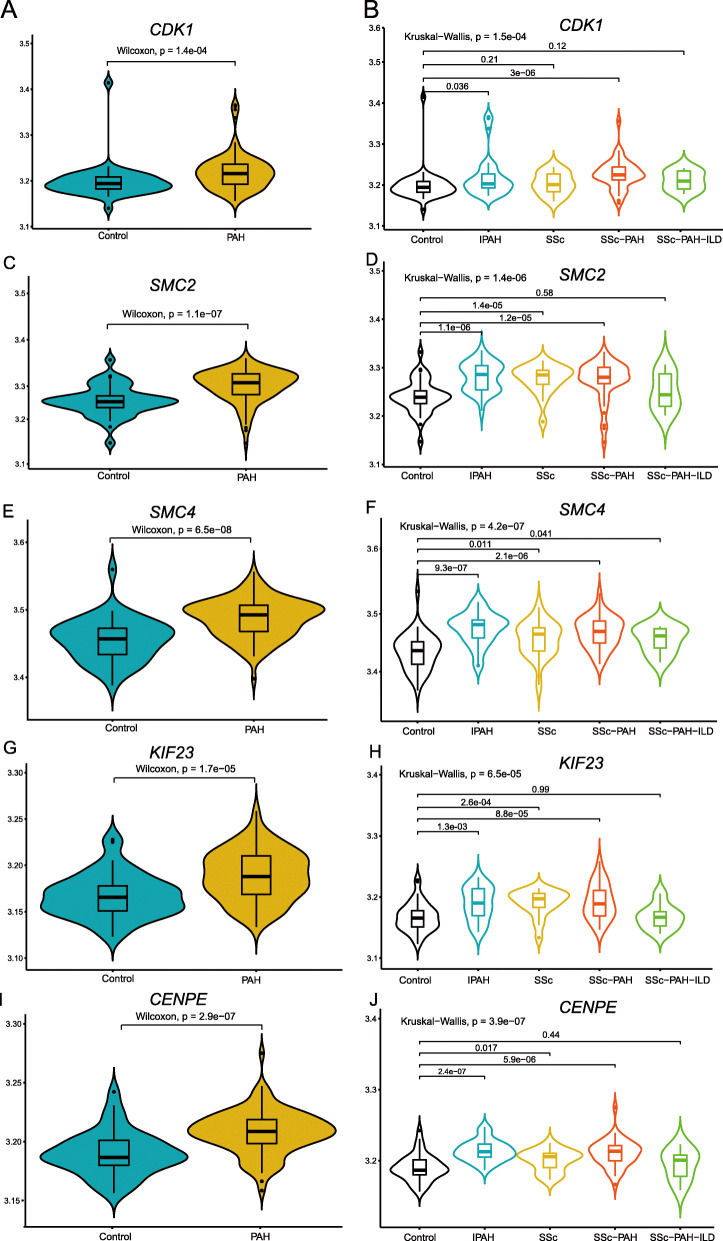
Violin diagram showing the expression levels of five hub genes are highly corrected with PAH, especially with IPAH and SSc-PAH. These hub genes include *CHEK1* (**a**-**b**), *SMC2* (**c**-**d**), *SMC4* (**e**-**f**), *KIF23* (**g**-**h**) and *CENPE* (**i**-**j**). *P*-values were respectively obtained from two-sample Wilcoxon test and multiple-sample Kruskal−Wallis test

To further explore the potential role of these hub genes in PAH, we used the transcriptomic data set from a cohort of PBMCs (GSE33463) to validate the nine hub genes. As shown in Fig. 4, five hub genes (*CDK1, SMC2, SMC4, KIF23* and *CENPE*) were further identified with significant increased expression in PAH patients compared to control (*p* = 0.00014 for *CDK1*, *p* = 1.1 × 10^− 7^ for *SMC2*, *p* = 6.5× 10^− 8^ for *SMC4*, *p* = 1.7× 10^− 5^ for *KIF23*, *p* = 2.9× 10^− 7^ for *CENPE*; Wilcoxon and Kruskal-Wallis test). Notably, we also found that the five hub genes have significant increased expression in several PAH subtypes compared to control, especially in IPAH and systemic sclerosis (SSc)-PAH for all five hub genes (Fig. 4). Moreover, except *CDK1*, there are 4 hub genes with increased expression in SSc compared to control. In addition, only *SMC4* showed the higher expression in SSc-PAH-ILD than control. For other four hub genes (*CDC6, CHEK1, TOP2A* and *PLK4*), there were no significant difference between PAH patients and control (Fig.S4).

**Fig. 5:**
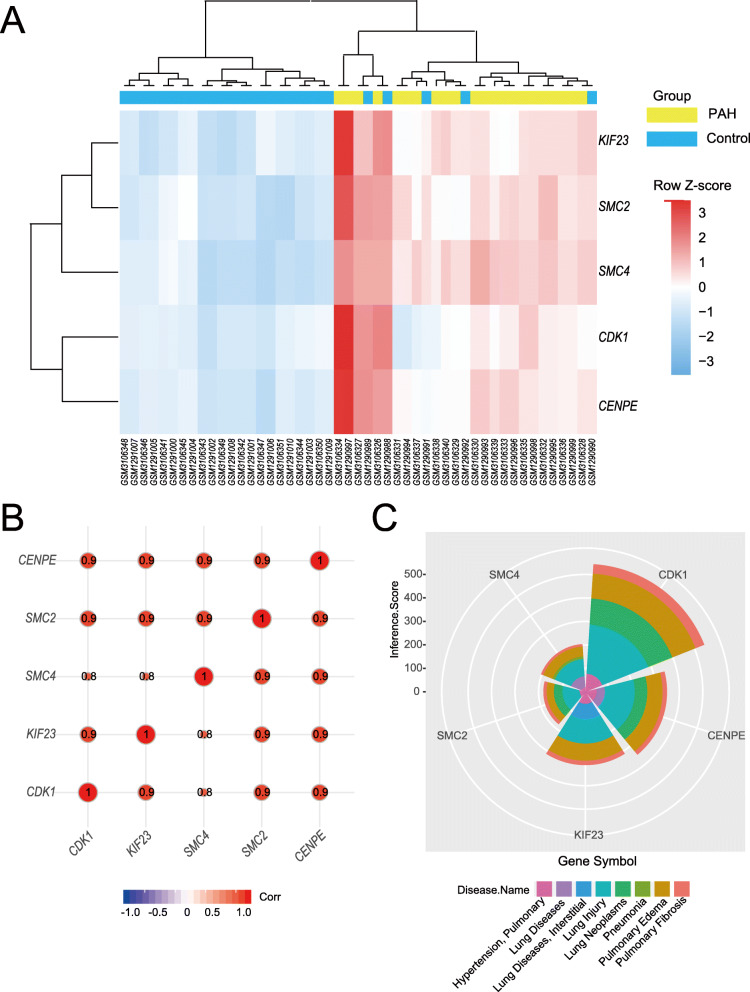
Functional association of five hub genes. **a**. Clustering heatmap of 5 hub genes. “Red” indicates high relative expression, and “Blue” indicates low relative expression*.***b***.* Correlations analysis of hub genes. “Red” represents positive correlation and “Blue” represents negative correlation. The size of circle and number indicates correlation coefficient. **c**. Pie Chart indicates distribution of respiratory systemic disease correlative with hub genes. Each module with color represents one respiratory disease and each area indicates inference scores between genes and diseases

To further confirm the significant difference between PAH cohorts and control cohorts, we made the cluster analysis which revealed up-regulation of hub genes in cases while down-regulation in control (Fig. 5a). Correlation analysis showed that the five hub genes have positive correlation between each other with correlation coefficient greater that 0.8 (Fig. 5b). Moreover, we also performed the association analysis between hub genes and respiratory tract diseases based on the Inference Score from CTD database. Although lung neoplasms covered the largest area with high inference score in all hub genes, other respiratory tract diseases, including hypertension pulmonary, lung diseases interstitial and pulmonary fibrosis, also presented a degree of association with hub genes (Fig. 5c).

### Prediction of miRNAs targeting hub genes and function analysis for miRNAs

**Fig. 6:**
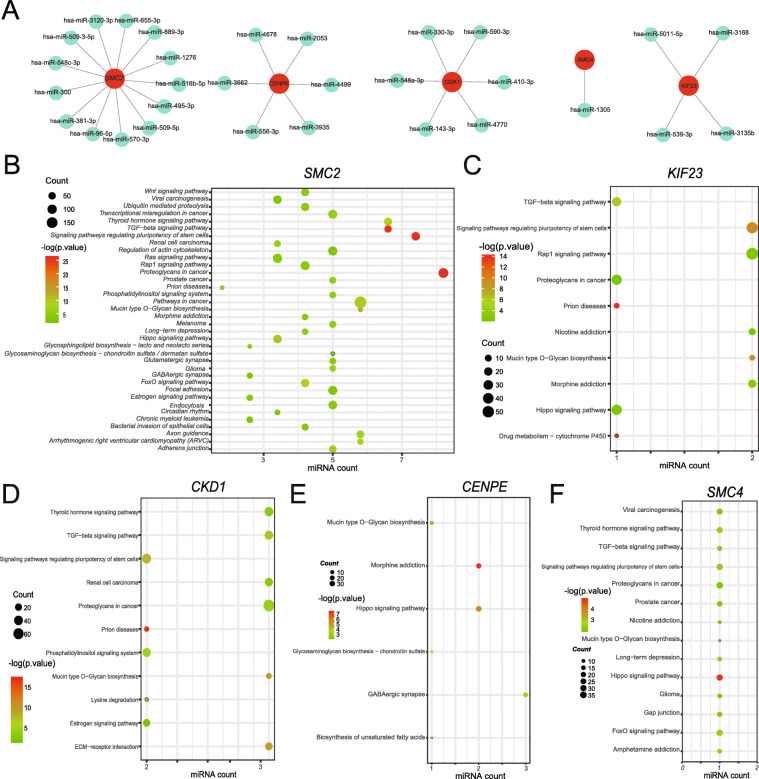
Functional prediction of miRNA targeting hub genes. **a***.* miRNA-mRNA network. The red represents mRNA of hub genes, green represents predicted miRNA. **b**-**f**. Bubble chart shows the KEGG pathway enrichment of each mRNA’s predicted miRNAs. The sizes of the dots indicate the number of genes and colors of dots indicate *p*-value

To further explore the potential role of miRNAs in PAH, we found 30 probable miRNAs that target these five hub genes based on the prediction of Diana-microT-CDS, miRDB, TargetScan and mirDIP (Fig. 6a). Among these miRNAs, that there were 13 miRNAs targeting SMC2, 6 miRNAs targeting CENPE, 6 miRNAs targeting CDK1, 4 miRNAs targeting KIF23 and one miRNA targeting SMC4 (Fig. 6a). Subsequently, using Diana-miRPath v3.0, we conducted KEGG pathway enrichment analysis that exhibited miRNAs majorly focus on TGF − beta signaling pathway, signaling pathways regulating pluripotency of stem cells, Proteoglycans in cancer, Mucin type O − Glycan biosynthesis and Hippo signaling pathway (Fig. 6b-f).

## Discussion

With the development of high-throughput gene detection technology, gene expression studies have been conducted to reveal the molecular mechanisms of the progression of PAH, but the specific genetic changes of PAH are still not clear. Herein, we extracted and merged data from GSE53408 and GSE113439 datasets containing gene expression profiles of both PAH and normal tissues, and identified a total of 521 DEGs after a series of preprocessing process. Functional annotation indicated that these DEGs were mainly involved in mitotic cell cycle and microtubule cytoskeleton organization. By constructing the PPI network and further modular analysis, we identified some key genes and predicted miRNAs targeting hub genes with function analysis, all of which can provide new insights into the pathogenesis of PAH.

It has been known that the pathogenesis of PAH is complex and uncertain including the increasing ratio of endothelium-derived vasoconstrictors to vasodilators [26], apoptosis resistance, and hyperproliferation of pulmonary artery vascular smooth muscle cells (PASMC) [27], increasing plasma serotonin levels [28] and decreased function of voltage-gated potassium channels in PASMC [29]. By integrated bioinformatical analysis, we identified the mitotic cell cycle process as the considerable biological process dealing with pathogenesis of PAH, which had been found as target sites for some drugs that could delay even overturn the progress of PAH through inhibiting the proliferation of vascular smooth muscle [22]. For an instance, Kentaroet et al. [30] indicated protein tyrosine kinases inhibitors inhibit multiple steps of the vascular SMC cell cycle and a progressive irreversible endothelial cell dysfunction induced by Monocrotaline pyrrole, leading to inactivation of *CDC2* kinase and irreversible cell-cycle arrest at the G2 checkpoint, which was ulteriorly certified by Thomas’s study [31]. Moreover, microtubule cytoskeleton organization had been reported to regulate the essential processes of smooth muscle cell migration through cell contraction and focal adhesion assembly, which was associated with lung and vascular diseases [23]. Further, Yunchao’s study documented that microtubule-active agents could influence the state of microtubule polymerization to modify the nitric oxide (NO) production in pulmonary artery endothelial cells (PAEC), which might well provide a promising avenue for the treatment of PAH [32].

In present studies, *CDK1*, also named *CDC2*, is one of Cyclin-dependent kinase family, which are essential conditioning agent of cell cycle progression [33]. More importantly, previous study had discovered the indispensable role of *CDK1* in G1/S and G2/M phase transitions of the eukaryotic cell cycle, as a vital catalytic subunit of M-phase promoting factor [34]. It has been reported that the phosphorylation and dephosphorylation process of proteins encoded by *CDK1* plays an essential role in regulating mitochondrial function, maintaining mitochondrial homeostasis and improving cell-cycle progression [35]. It had been reported that PASMC mitochondria play an essential role in the process of PAH by regulating energy metabolism including glucose oxidation and increased cytoplasmic glycolysis represent, which were associated with oxidative stress mechanism in hypoxic microenvironment [36]. Interestingly, recent studies have demonstrated an important interaction between the mitochondrial cycle and the cell cycle, leading to increased proliferation in human PAH PASMCs, which also verifies the interaction between cell cycle, especially mitochondrial cycle and PAH [24]. Although most studies considered abnormal expression of *CDK1* as risk factors for a variety of tumors, such as hepatocellular, breast and colorectal [37, 38], several researches had found that *CDK1* participated in molecular mechanism of PAH, mainly influencing mitochondrial dynamics [39].

Similarly, *SMC2* and *SMC4* are two vital core subunits of condensin, which plays an essential role in mitotic chromosome condensation [40]. Recently, it has been reported that the condensin complex also play role in DNA repair and transcriptional regulation during interphase. More importantly, Yoko’s study has found that *SMC2* was transcriptionally regulated by *MYCN*, a carcinogenic gene with poor prognosis in *MYCN*-amplified neuroblastoma cells [41]. There was also evidence indicating the transcription of *SMC4* was activated by NF-κB through regulation of miR-16 and miR-21 in gastric cancer, and *SMC4* could participate in chronic lymphocytic leukemia (CLL) with post-transcriptional regulation of miR-15/− 16 family members [42]. Therefore, *SMC2* and *SMC4* might function in the regulation of cell proliferation based on aforementioned mechanisms, which was further confirmed by Verónica’s study that indicated that *SMC2* transcription was directly activated by WNT signaling, as a key player in the mitotic cell division machinery [43].

*KIF23*, as both a regulator of cytokinesis and a motor enzyme of microtubules, is critical for the microtubule bundling during cytokinesis [44]. In addition, *KIF23* expression was regulated in cell cycle with peaks in G2/M phase, indicating the role of *KIF23* in boosting cell hyperplasia [45]. Based on above features, up-regulated *KIF23* has been considered as one of biomarkers in multiple tumors including lung cancer, malignant pleural mesothelioma, glioma and hepatocellular carcinoma [46, 47].

*CENPE*, the largest kinesin of Kinesins families, has been found with roles in microtubule kinetochore capture, which contributes to chromosome congression and alignment [48]. Sullivan’s study [49] also demonstrated *CENP-C* and *CENP-E* are necessary components of functional centromeres in human. Through traveling unidirectionally along microtubule tracks, *CENPE* participated in intracellular transport or cell division, which interpreted its role in cell cycle in our study [50]. It also had been reported that CENPE was highly expressed in the G2/M phase of the cell cycle and promoted lung adenocarcinoma (LUAD) proliferation, regulated by *FOXM1* in previous study [51]. In addition, several studies had considered *CENPE* as drug targets and even entered Phase I and II clinical trials for threat of certain tumors [52]. It is known to all that PAH is recognized as the most common complication of systemic sclerosis (SSc) due to severe vascular lesion. Notably, *CENPE* was increased in vascular progenitors and mature endothelial cells as one of the scleroderma autoantigens with *IFI-16* in McMahan’s study [53], which supported its role in proliferation of vascular endothelial cells in our study.

In this study, we found that there was significant enrichment of DEGs in cell cycle associated with PAH through vascular smooth muscle cells proliferation. Notably, KEGG pathways analysis of predicted miRNAs also suggest similar potential biological mechanism for PAH. It had been reported that *BMPR-II*, a receptor of TGF-beta family, participated in formation of PAH by germline mutations [54, 55]. Through text mining, Goumans’s [56] study had also affirmed that TGF-β/ALK1 signaling could stimulate endothelial cells (EC) migration, proliferation and tube formation by inducing Smad1/5 activation, which played an essential role in vascular dysfunction [57]. Moreover, Hippo signaling pathway [58] was reported as a master regulator of proliferation and apoptosis balance with function of regulating cell proliferation and inducing cell differentiation or apoptosis. However, excessive activation of Hippo signaling pathway by its major reciprocal effectors Yap/Taz would promote proliferation via regulating other transcriptional factors, as significant component of PAH progression in Tatiana V’s research [59]. All of these studies supported our hypothesis on hub genes may participate in PAH through promoting biological process of vascular proliferation. Moreover, our study also found some potential pathways such as Signaling pathways regulating pluripotency of stem cells, which might provide new opinions on mechanism or therapeutic targets for PAH. Significantly, several studies have started to focus on implantation of Mesenchymal Stem Cells in postponing or improving the process of cardiovascular diseases including PAH, which also gives us several inspiration on novel therapeutic intervention for PAH [60].

However, there still are some limitations in this study. To reach a solid correlation between hub genes and PAH, further validation of enlarged samples would be necessary. Given the shortage of silicon analyses and some dataset validation, a series of in-depth experiments in vitro and vivo are needed in the future to confirm the specific functions of these genes in PAH and detailed pathway regulation.

## Conclusion

In conclusion, a total of 432 up-regulated DEGs and 89 down-regulated DEGs were identified in PAH samples. The DEGs related to the up-regulation of cell cycle process (*CDK1, SMC2, SMC4, KIF23, CENPE*), which may activate the proliferation of PASM in the process of PAH. The predicted miRNAs were found enriched in TGF-β and Hippo signaling pathway. These findings are expected to a get a further insight into biomarkers for PAH diagnosis and molecular mechanisms of PAH pathogenesis.

## Supplementary information

**Additional file 1 Figure S1.** The summary and description of the study workflow.

**Additional file 2 Figure S2 A.** Box diagram showes the homogeneous and comparable distribution of expression profile for each sample. The horizontal ordinate represents samples and the ordinate represents expression distribution of each sample. The green and red rectangles represent the case or control groups, respectively. **B.** Module 2 with MCODE score of 19.789 and Module 3 with MCODE score of 11.125 from the PPI network. The color shadow of nodes represents node’s Mcode_score (degree of connection of nodes).

**Additional file 3 Figure S3** Protein-protein interaction networks of the 521 DEGs using online database STRING. Cycle nodes represent genes and the thickness of edges between nodes represent the combined score.

**Additional file 4 Figure S4** Violin diagrams show the expression levels of eliminated genes without significant difference between PAH and control (*P* > 0.05). *P*-values were obtained from two-sample Wilcoxon test and multiple samples Kruskal−Wallis test, respectively.

**Additional file 5 Table S1**. Characteristics of the individual studies. **Table S2**. List of the top 100 DEGs according to the rank of adjusted *P* value (adj. *P*-value). **Table S3**. The enriched gene ontology (GO) categories of differentially expressed genes (DEGs). **Table S4**. The significantly Top 20 enriched pathways of differentially expressed genes (DEGs). **Table S5**. List of hub genes by PPI Degree and Modular Analysis. **Table S6**. Pathway enrichment analysis of Module genes function. **Table S7**. List of genes representing the top 50 key genes in more than 6 ways*.*

## Data Availability

All the data generated or analyzed in this study have been included in this published article and its supplementary Table and Figure files. The raw matrix datasets can be downloaded from the NCBI-GEO database by searching “GSE53408” (https://www.ncbi.nlm.nih.gov/geo/query/acc.cgi?acc=GSE53408), “GSE113439” (https://www.ncbi.nlm.nih.gov/geo/query/acc.cgi?acc=GSE113439) and “GSE33463” (https://www.ncbi.nlm.nih.gov/geo/query/acc.cgi?acc=GSE33463). GPL6244 annotation files can be required from the website: https://www.ncbi.nlm.nih.gov/geo/query/acc.cgi?acc=GPL6244.

## Abbreviations

PAH: Pulmonary arterial hypertension
DEGs: Differentially expressed genes
PPI: Protein-protein interaction
miRNAs: Micro-RNAs
mPAP: Mean pulmonary arterial pressure
PCWP: Pulmonary capillary wedge pressure
WSPH: World symposium on pulmonary hypertension
RV: Right ventricle
IPAH: Idiopathic pulmonary arterial hypertension
Log2FC: Log2 fold change
PBMCs: Peripheral blood monouclear cells
CTD: Comparative toxicogenomics database
GO: Gene ontology
BP: Biological process
CC: Cell component
MF: Molecular function
STRING: The search tool for the retrieval of interacting genes
rRNA: Ribosomal RNA
SSc: Systemic sclerosis
PASMC: Pulmonary artery vascular smooth muscle cells
NO: Nitric oxide
PAEC: Pulmonary artery endothelial cells
CLL: Chronic lymphocytic leukemia
LUAD: Lung adenocarcinoma
EC: Endothelial cells
