# Supplementary figures and images for: Integrative analyses of gene expression profile reveal potential crucial roles of mitotic cell cycle and microtubule cytoskeleton in pulmonary artery hypertension
Source: BMC Med Genomics. 2020 Jun 26;13:86. doi: 10.1186/s12920-020-00740-x (PMC7318763; doi:10.1186/s12920-020-00740-x)

Supplementary Figure 1

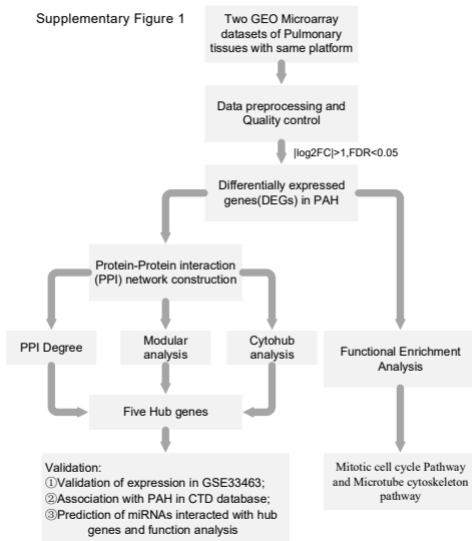

Supplement: Supplementary file 1 — Additional file 1 Figure S1. The summary and description of the study workflow. [file 12920_2020_740_MOESM1_ESM.pdf]

Supplementary Figure 4

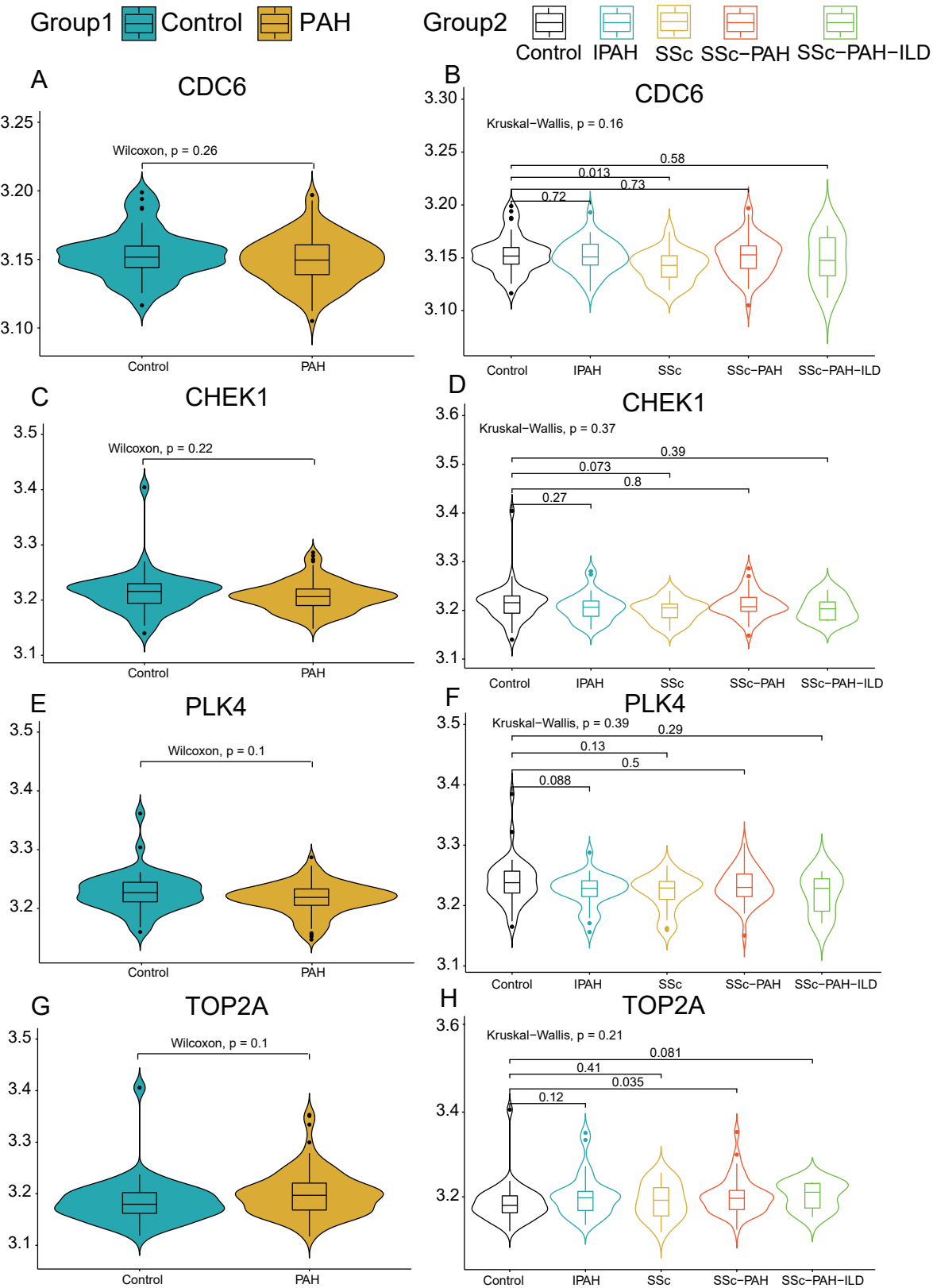

Supplement: Supplementary file 4 — Additional file 4 Figure S4 Violin diagrams show the expression levels of eliminated genes without significant difference between PAH and control (P > 0.05). P-values were obtained from two-sample Wilcoxon test and multiple samples Kruskal−Wallis test, respectively. [file 12920_2020_740_MOESM4_ESM.pdf]
