# Supplementary material for: Integrative analyses of gene expression profile reveal potential crucial roles of mitotic cell cycle and microtubule cytoskeleton in pulmonary artery hypertension
Source: BMC Med Genomics. 2020 Jun 26;13:86. doi: 10.1186/s12920-020-00740-x (PMC7318763; doi:10.1186/s12920-020-00740-x)
Supplement: Supplementary file 5 — Additional file 5 Table S1. Characteristics of the individual studies. Table S2. List of the top 100 DEGs according to the rank of adjusted P value (adj. P-value). Table S3. The enriched gene ontology (GO) categories of differentially expressed genes (DEGs). Table S4. The significantly Top 20 enriched pathways of differentially expressed genes (DEGs). Table S5. List of hub genes by PPI Degree and Modular Analysis. Table S6. Pathway enrichment analysis of Module genes function. Table S7. List of genes representing the top 50 key genes in more than 6 ways. [file 12920_2020_740_MOESM5_ESM.docx]

| **Table S 1. Characteristics of the individual studies.** | | | | | | |
| --- | --- | --- | --- | --- | --- | --- |
| **GEO ID** | **Patients/**  **Control** | **Platform** | **Sample source** | **Country** | **Years** | **Reference** |
| GSE53408 | 12/11 | \| [GPL6244](https://www.ncbi.nlm.nih.gov/geo/query/acc.cgi?acc=GPL6244) \| [HuGene-1_0-st] Affymetrix Human Gene 1.0 ST Array \| \| --- \| --- \| | [Homo sapiens](https://www.ncbi.nlm.nih.gov/Taxonomy/Browser/wwwtax.cgi?mode=Info&id=9606) | Canada | 2013, 2018 | Zhao Y et al. (2014) |
| GSE113439 | 15/11 | \| [GPL6244](https://www.ncbi.nlm.nih.gov/geo/query/acc.cgi?acc=GPL6244) \| [HuGene-1_0-st] Affymetrix Human Gene 1.0 ST Array \| \| --- \| --- \| | [Homo sapiens](https://www.ncbi.nlm.nih.gov/Taxonomy/Browser/wwwtax.cgi?mode=Info&id=9606) | Canada | 2018 | None |
| GSE33463 | 99/41 | \| GPL6947 \| Illumina HumanHT-12 V3.0 expression beadchip \| \| --- \| --- \| | [Homo sapiens](https://www.ncbi.nlm.nih.gov/Taxonomy/Browser/wwwtax.cgi?mode=Info&id=9606) | USA | 2012 | Cheadle et al. (2012) |

**Table S 2. List of the top 100 DEGs according to the rank of adjusted *P* value (adj.*P*-value)**

| **Gene Symbol** | **adj.*P*-value** | **Gene**  **Symbol** | **adj.*P*-value** | **Gene**  **Symbol** | **adj.*P*-value** |
| --- | --- | --- | --- | --- | --- |
| SLC4A1AP | 1.96E-21 | GPR146 | 6.29E-18 | IMPA1 | 6.04E-17 |
| USP16 | 5.93E-21 | SMC3 | 6.58E-18 | RAPGEF6 | 6.10E-17 |
| GALNT1 | 1.44E-20 | GOLGA4 | 7.68E-18 | COPB1 | 7.13E-17 |
| CCDC59 | 2.29E-20 | ZNF28 | 7.87E-18 | RSRC1 | 7.34E-17 |
| NSRP1 | 3.02E-20 | GCC2 | 8.46E-18 | IDE | 8.10E-17 |
| USP8 | 3.27E-20 | ZFX | 8.85E-18 | DLD | 8.48E-17 |
| SCARNA4 | 7.18E-20 | SMC6 | 1.09E-17 | TAX1BP1 | 8.63E-17 |
| ROCK2 | 1.85E-19 | EPS8 | 1.17E-17 | BOD1L1 | 9.03E-17 |
| ROCK1 | 2.03E-19 | BLZF1 | 1.24E-17 | ZNF654 | 9.04E-17 |
| USO1 | 3.20E-19 | ICAM2 | 1.24E-17 | PYROXD1 | 9.11E-17 |
| HOOK3 | 3.20E-19 | PNN | 1.26E-17 | NOL8 | 9.17E-17 |
| NBN | 3.79E-19 | CDC5L | 1.28E-17 | THOC2 | 9.57E-17 |
| KIF5B | 4.32E-19 | CEP350 | 1.29E-17 | TXLNG | 9.91E-17 |
| WRN | 6.27E-19 | ATP6V1C1 | 1.45E-17 | SNORD74 | 1.09E-16 |
| RANBP2 | 1.09E-18 | OPA1 | 1.54E-17 | N4BP2 | 1.18E-16 |
| COPS2 | 1.13E-18 | CRIP2 | 1.54E-17 | EIF3A | 1.22E-16 |
| ZC3H13 | 1.16E-18 | WFS1 | 1.56E-17 | CWC22 | 1.24E-16 |
| DNAJC21 | 1.16E-18 | DIAPH2 | 1.70E-17 | FXR1 | 1.25E-16 |
| GOLGB1 | 1.41E-18 | DNM1L | 1.72E-17 | CCDC88A | 1.40E-16 |
| OSBPL8 | 1.41E-18 | ZNF33A | 1.72E-17 | ANKRD12 | 1.43E-16 |
| SMC4 | 1.69E-18 | FRS2 | 1.72E-17 | RSL1D1 | 1.45E-16 |
| RPAP3 | 1.69E-18 | LARP7 | 1.78E-17 | RANBP6 | 1.47E-16 |

**Table S3. The enriched gene ontology (GO) categories of differentially expressed genes (DEGs)**

| **Category** | **GO-Term** | **Count** | **adj.*P*-value** |
| --- | --- | --- | --- |
| BP | GO:0000278-mitotic cell cycle | 76 | 7.18E-10 |
| BP | GO:1903047-mitotic cell cycle process | 66 | 3.46E-09 |
| BP | GO:0000226-microtubule cytoskeleton organization | 48 | 1.05E-08 |
| BP | GO:0140014-mitotic nuclear division | 34 | 1.81E-08 |
| BP | GO:0022402-cell cycle process | 83 | 2.80E-07 |
| CC | GO:0043232-intracellular non-membrane-bounded organelle | 204 | 3.07E-13 |
| CC | GO:0031981-nuclear lumen | 196 | 4.50E-11 |
| CC | GO:0015630-microtubule cytoskeleton | 81 | 5.07E-10 |
| CC | GO:0005815-microtubule organizing center | 56 | 7.23E-08 |
| CC | GO:0005856-cytoskeleton | 115 | 9.16E-08 |
| MF | GO:0005524-ATP binding | 104 | 1.73E-13 |
| MF | GO:0032559-adenyl ribonucleotide binding | 105 | 1.24E-12 |
| MF | GO:0030554-adenyl nucleotide binding | 105 | 1.72E-12 |
| MF | GO:0035639-purine ribonucleoside triphosphate binding | 114 | 9.49E-12 |
| MF | GO:0043168-anion binding | 151 | 1.33E-11 |

Note: GO, gene ontology; BP, biological process; CC, cellular component；MF, molecular function; adj.*P*-value < 0.05 was considered as the threshold of significant difference.

**Table S4. The significantly Top 20 enriched pathways of differentially expressed genes (DEGs)**

| **Pathway** | **ID** | **Term** | **Count** | **FDR** |
| --- | --- | --- | --- | --- |
| Reactome | R-HSA-1640170 | Cell Cycle | 45 | 6.16E-17 |
| Reactome | R-HSA-392499 | Metabolism of proteins | 61 | 1.55E-13 |
| Reactome | R-HSA-69278 | Cell Cycle, Mitotic | 36 | 3.44E-13 |
| Reactome | R-HSA-74160 | Gene Expression | 66 | 3.01E-12 |
| Reactome | R-HSA-5653656 | Vesicle-mediated transport | 34 | 2.03E-10 |
| Reactome | R-HSA-6811442 | Intra-Golgi and retrograde Golgi-to-ER traffic | 20 | 1.30E-09 |
| Reactome | R-HSA-597592 | Post-translational protein modification | 39 | 3.77E-09 |
| Reactome | R-HSA-199991 | Membrane Trafficking | 30 | 1.16E-08 |
| Reactome | R-HSA-68886 | M Phase | 23 | 1.22E-08 |
| Reactome | R-HSA-109582 | Hemostasis | 30 | 1.36E-07 |
| Reactome | R-HSA-68877 | Mitotic Prometaphase | 14 | 3.39E-07 |
| Reactome | R-HSA-8856688 | Golgi-to-ER retrograde transport | 14 | 3.76E-07 |
| Reactome | R-HSA-6811434 | COPI-dependent Golgi-to-ER retrograde traffic | 12 | 1.53E-06 |
| Reactome | R-HSA-195258 | RHO GTPase Effectors | 19 | 3.93E-06 |
| Reactome | R-HSA-3108232 | SUMO E3 ligases SUMOylate target proteins | 11 | 7.99E-06 |
| Reactome | R-HSA-162582 | Signal Transduction | 64 | 8.08E-06 |
| Reactome | R-HSA-2990846 | SUMOylation | 11 | 1.15E-05 |
| Reactome | R-HSA-168256 | Immune System | 47 | 1.36E-05 |
| Reactome | R-HSA-194315 | Signaling by Rho GTPases | 21 | 1.74E-05 |
| Reactome | R-HSA-5685942 | HDR through Homologous Recombination (HRR) | 9 | 3.35E-05 |

Note: Pathways with corrected P value less than 0.05 were considered significantly enriched by differential expressed genes

**Table S5. List of hub genes by PPI Degree and Modular Analysis**

| GENE  Symbol | Degree | GENE  Symbol | MCODE score | GENE  Symbol | MCODE score |
| --- | --- | --- | --- | --- | --- |
| **TOP2A** | 100 | **SMC4** | 24 | PLK4 | 24 |
| **TOP2B** | 81 | **SMC2** | 24 | **TOP2A** | 24 |
| **CDK1** | 61 | NCAPG | 24 | KIF18A | 24 |
| **LRRK2** | 57 | **KIF11** | 24 | ASPM | 24 |
| **HSP90AA1** | 53 | **KIF23** | 24 | NUSAP1 | 24 |
| **EPRS** | 52 | CENPF | 24 | CEP55 | 24 |
| **POLR2B** | 50 | CENPE | 24 | DLGAP5 | 24 |
| **SMC2** | 48 | KIF20A | 24 |  |  |
| **CHEK1** | 47 | **CDK1** | 24 |  |  |
| **SMC4** | 45 | TPX2 | 24 |  |  |
| **KIF23** | 44 | ECT2 | 24 |  |  |
| **PLK4** | 44 | **CDC6** | 24 |  |  |
| **KIF11** | 43 | **CHEK1** | 24 |  |  |
| **CDC6** | 41 | PRC1 | 24 |  |  |
| **CENPE** | 40 | MLF1IP | 24 |  |  |

**Table S6. Pathway enrichment analysis of Module genes function.**

| **Pathway** | **ID** | **Term** | **Count** | **FDR** |
| --- | --- | --- | --- | --- |
| **Module1** |  |  |  |  |
| Reactome | R-HSA-1640170 | Cell Cycle | 15 | 1.38E-17 |
| Reactome | R-HSA-69278 | Cell Cycle, Mitotic | 14 | 3.18E-17 |
| Reactome | R-HSA-68886 | M Phase | 10 | 1.51E-12 |
| Reactome | R-HSA-68877 | Mitotic Prometaphase | 8 | 2.62E-12 |
| Reactome | R-HSA-983189 | Kinesins | 6 | 2.78E-10 |
| Reactome | R-HSA-6811434 | COPI-dependent Golgi-to-ER retrograde traffic | 6 | 3.93E-09 |
| Reactome | R-HSA-2514853 | Condensation of Prometaphase Chromosomes | 4 | 8.19E-09 |
| Reactome | R-HSA-8856688 | Golgi-to-ER retrograde transport | 6 | 1.33E-08 |
| Reactome | R-HSA-983231 | Factors involved in megakaryocyte development and platelet production | 6 | 6.92E-08 |
| Reactome | R-HSA-6811442 | Intra-Golgi and retrograde Golgi-to-ER traffic | 6 | 1.33E-07 |
| **Module2** |  |  |  |  |
| Reactome | R-HSA-6791226 | Major pathway of rRNA processing in the nucleolus and cytosol | 9 | 4.81E-16 |
| KEGG | hsa03008 | Ribosome biogenesis in eukaryotes | 8 | 4.81E-16 |
| Reactome | R-HSA-8868773 | rRNA processing in the nucleus and cytosol | 9 | 5.10E-16 |
| Reactome | R-HSA-72312 | rRNA processing | 9 | 6.07E-16 |
| Reactome | R-HSA-6790901 | rRNA modification in the nucleus and cytosol | 7 | 3.86E-15 |
| Reactome | R-HSA-74160 | Gene Expression | 10 | 3.53E-09 |
| **Module3** |  |  |  |  |
| Reactome | R-HSA-3371556 | Cellular response to heat stress | 7 | 1.32E-12 |
| Reactome | R-HSA-3371453 | Regulation of HSF1-mediated heat shock response | 6 | 3.92E-11 |
| KEGG | hsa04141 | Protein processing in endoplasmic reticulum | 6 | 5.87E-09 |
| Reactome | R-HSA-2262752 | Cellular responses to stress | 7 | 9.78E-09 |
| Reactome | R-HSA-392499 | Metabolism of proteins | 9 | 6.93E-08 |
| Reactome | R-HSA-3108232 | SUMO E3 ligases SUMOylate target proteins | 4 | 3.18E-06 |
| Reactome | R-HSA-2990846 | SUMOylation | 4 | 3.34E-06 |
| Reactome | R-HSA-168273 | Influenza Viral RNA Transcription and Replication | 4 | 9.01E-06 |
| Reactome | R-HSA-168255 | Influenza Life Cycle | 4 | 1.01E-05 |
| Reactome | R-HSA-168254 | Influenza Infection | 4 | 1.22E-05 |
| Reactome | R-HSA-69278 | Cell Cycle, Mitotic | 5 | 4.08E-05 |
| Reactome | R-HSA-3108214 | SUMOylation of DNA damage response and repair proteins | 3 | 8.15E-05 |
| Reactome | R-HSA-1640170 | Cell Cycle | 5 | 8.48E-05 |

**Table S7. List of genes representing the top 50 key genes in more than 6 ways*.***

| GENE  Symbol | A total of ways for selecting the gene | GENE  Symbol | A total of ways for selecting the gene | GENE  Symbol | A total of ways for selecting the gene |
| --- | --- | --- | --- | --- | --- |
| KIF23 | 11 | EPRS | 9 | CKAP5 | 7 |
| **PLK4** | 10 | HIST2H2AC | 9 | CEP55 | 7 |
| CDC6 | 10 | HSP90AA1 | 9 | TPX2 | 7 |
| CDK1 | 10 | **TOP2A** | 9 | **SMC2** | 7 |
| **DDX18** | 10 | NOP58 | 9 | CENPE | 7 |
| **DDX21** | 10 | WDR3 | 8 | ATAD2 | 7 |
| **HSPD1** | 9 | LRRK2 | 7 | KIF18A | 7 |
| **TOP2B** | 9 | DNAJC10 | 7 | **SMC4** | 7 |
| **POLR2B** | 9 | CDC5L | 7 | **CHEK1** | 7 |
| **RANBP2** | 9 | DHX9 | 7 |  |  |

Note: The total 12 ways: Betweenness, BottleNeck, Closeness, ClusteringCoefficient, Degree, DMNC, EcCentricity, EPC, MCC, MNC, Radiality, Stress.
